# Effect of five years of biannual azithromycin mass drug administration on enteric fever seroincidence in Niger: A randomized control trial

**DOI:** 10.64898/2026.08.03.26359544

**Published:** 2026-08-04

**Authors:** Kristen Aiemjoy, Jessica C Seidman, Ahmed M Arzika, Ramatou Maliki, Amza Abdou, Douglas Ezra Morrison, Kristina W Lai, Denise O. Garrett, Claire Munroe, Abel Gonzalez, Leah Sukri, Elodie Lebas, Catherine Cook, Benjamin F Arnold, Thomas M Lietman, Kathleen M Neuzil, Jason R Andrews, Jeremy D Keenan, Richelle C Charles

## Abstract

**Background:**

Enteric fever, caused by *Salmonella enterica* serovars Typhi and Paratyphi, remains a major cause of morbidity in low- and middle-income countries, yet burden estimation is challenging in regions without blood culture surveillance. Azithromycin is effective for treating enteric fever, and mass drug administration (MDA) to young children reduces all-cause mortality in sub-Saharan Africa, but its impact on community-level enteric fever burden is unknown. We assessed enteric fever seroincidence in rural Niger and evaluated the effect of biannual azithromycin MDA affected.

**Principal Findings:**

This was a secondary analysis of the MORDOR trial ( NCT02048007 ), a cluster-randomized, placebo-controlled study in 30 communities in Dosso Region, Niger. Children aged 1–59 months received biannual azithromycin or placebo. Dried blood spots collected at baseline (2015) and Year 5 (2020) were tested for IgA and IgG antibodies against Hemolysin E using kinetic ELISA. Seroincidence was estimated using maximum likelihood methods based on antibody kinetics from blood culture-confirmed enteric fever patients. Samples from 1,417 children were analyzed (423 baseline; 994 Year 5). Overall seroincidence approximately doubled from baseline to Year 5, increasing from 68.5 (95% CI 58.0–80.7) to 145.2 (95% CI 132.3–159.3) per 100 person-years in placebo communities and from 74.7 (95% CI 56.5–98.7) to 135.2 (95% CI 112.4–162.6) in azithromycin communities, with no significant difference between arms (difference-in-differences −16.2; 95% CI -53.1-20.8 ). Among children <2 years, the increase in seroincidence was smaller in the azithromycin arm (37.5; 95% CI: 1.3-73.6) compared to placebo (81.1; 95% CI 54.0-108.2), with a difference-in-differences of −43.7 per 100 person-years (95% CI −88.8 - 1.5 to 0.02).

**Significance:**

Enteric fever seroincidence among young children in rural Niger is high and increased substantially over the five-year study period. Biannual azithromycin MDA did not significantly reduce overall seroincidence, though a potential attenuation was observed among children under 2 years of age. These findings underscore the importance of Niger’s recent typhoid conjugate vaccine introduction and highlight the value of serosurveillance for monitoring enteric fever infection intensity in settings without blood culture surveillance.

## Full Text Availability

The license terms selected by the author(s) for this preprint version do not permit archiving in PMC. The full text is available from the preprint server.

